# Chemotaxis of *Escherichia coli* to major hormones and polyamines present in human gut

**DOI:** 10.1038/s41396-018-0227-5

**Published:** 2018-07-11

**Authors:** Joana G. Lopes, Victor Sourjik

**Affiliations:** Max Planck Institute for Terrestrial Microbiology and LOEWE Center for Synthetic Microbiology (SYNMIKRO), Marburg, Germany

## Abstract

The microorganisms in the gastrointestinal (GI) tract can influence the metabolism, immunity, and behavior of animal hosts. Increasing evidence suggests that communication between the host and the microbiome also occurs in the opposite direction, with hormones and other host-secreted compounds being sensed by microorganisms. Here, we addressed one key aspect of the host–microbe communication by studying chemotaxis of a model commensal bacterium, *Escherichia coli*, to several compounds present abundantly in the GI tract, namely catecholamines, thyroid hormones, and polyamines. Our results show that *E. coli* reacts to five out of ten analyzed chemicals, sensing melatonin, and spermidine as chemorepellents and showing mixed responses to dopamine, norepinephrine and 3,4-dihydroxymandelic acid. The strongest repellent response was observed for the polyamine spermidine, and we demonstrate that this response involves the low-abundance chemoreceptor Trg and the periplasmic binding protein PotD of the spermidine uptake system. The chemotactic effects of the tested compounds apparently correlate with their influence on growth and their stability in the GI tract, pointing to the specificity of the observed behavior. We hypothesize that the repellent responses observed at high concentrations of chemoeffective compounds might enable bacteria to avoid harmful levels of hormones and polyamines in the gut and, more generally, antimicrobial activities of the mucous layer.

## Introduction

Humans and other animals share a mutualistic relationship with numerous resident microorganisms, collectively known as the microbiome. Over the past two decades, the role of the host–microbiome interactions in a number of physiological processes became increasingly clear [1–3], and it is likely that the gut microorganisms evolved specific mechanisms to detect multiple compounds that are released into the lumen of the gastrointestinal (GI) tract by the endocrine and immune systems of the host [4–7].

Homeostasis of the mucous layer of intestinal epithelial cells (IECs) in the mammalian GI tract is regulated by a variety of signals including cellular polyamines and hormonal signals [8–10]. Due to the abundance of catecholamines in the GI tract, most gut-brain axis studies have focused on the interactions between gut bacteria and the most abundant catecholamines – epinephrine, norepinephrine (NE; also known as noradrenaline, NA) and dopamine [11–14]. Recently, more attention has also been drawn to the thyroid hormones, mainly serotonin and melatonin (5-methoxy-N-acetyltryptamine), that are synthesized from tryptophan in the IECs and involved in the regulation of the GI tract function and of circadian cycles [15–19]. Moreover, polyamines such as putrescine and spermidine may likewise have a role in microbial endocrinology [10]. Polyamines are introduced with the diet, but also produced by microorganisms and host cells and regulate many distinct cellular functions in eukaryotes and prokaryotes, which include proliferation and differentiation of intestinal cells [20, 21]. The concentrations of polyamines in the GI lumen can approach millimolar levels, and they are known to affect the microbiome composition [10, 22]. Enteric bacteria, such as *Escherichia coli*, possess specific systems for polyamine uptake [23, 24].

Chemotaxis is one way in which bacteria could react to the compounds present in the gut. It allows bacteria to navigate in environmental gradients of various chemicals [25–27] in order to locate conditions that are beneficial for growth [28]. Chemotaxis to self-secreted signals can also mediate collective behaviors, such as the autoinducer-2 dependent autoaggregation and biofilm formation by *E. coli* [29, 30]. The chemotactic signal transduction pathway of *E. coli* includes two high-abundance (or major) receptors, Tsr and Tar, as well as three low-abundance (or minor) receptors, Trg, Tap, and Aer. These receptors form mixed complexes in the membrane together with the histidine kinase CheA, where the autophosphorylation activity of CheA is inhibited by the increased exposure to attractants. Low-kinase activity leads to reduced phosphorylation of the motor regulator CheY, which promotes counter-clockwise (CCW) flagellar rotation and thus smooth swimming up the attractant gradient. In contrast, the exposure to repellents activates CheA and elevates CheY phosphorylation, inducing the clockwise (CW) rotation and swimming reorientation. Dephosphorylation of CheY is catalyzed by the phosphatase CheZ, which is essential to quickly readjust bacterial behavior. Additionally, the chemotaxis pathway includes an adaptation system that gradually offsets the initial stimulation by attractants or repellents through changes in receptor methylation.

Several previous studies have suggested that the animal pathogens *Helicobacter pylori*, *Campylobacter jejuni*, and *Salmonella enterica* exhibit chemotaxis toward several compounds derived from the human gastric epithelium [31–35] and that such chemotaxis plays an important role in bacterial invasion, as well as survival in the intestine [36–38]. Moreover, both commensal *E. coli* K-12 and enterohemorrhagic *E. coli* (EHEC) can sense NE as a chemoattractant [39–41]. This response requires conversion of NE to 3,4-dihydroxymandelic acid (DHMA) by the monoamine oxidase TynA and the aromatic aldehyde dehydrogenase FeaB of *E. coli*, with DHMA then serving as the chemoattractant recognized by Tsr in the nanomolar concentration range [40–42]. These results indicate that hormone taxis might be common among enteric bacteria and not limited to pathogens, but the scope of this behavior remained unclear.

The goal of this study was therefore to systematically investigate the extent to which *E. coli*—as a model commensal bacterium with a well-studied chemotaxis system—can exhibit taxis toward different catecholamines, thyroid hormones and polyamines. Our results demonstrate that *E. coli* responds, using both low and high abundance receptors, to a wide range of the physiologically important compounds that are known to accumulate in the gut lumen, including dopamine, NE, 3,4-dihydroxymandelic acid (DHMA), melatonin and spermidine, but not to their metabolic precursors. Notably, we show that the same compounds also affect growth of *E. coli*, in an apparent correlation with its chemotactic preferences. We therefore propose that the observed chemotactic responses may be used by commensal and pathogenic *E. coli* to locate optimal growth niches in the gut lumen, but also to avoid the antimicrobial activity of the mucous layer of the gut.

## Results

### Chemotactic response of the wild type *E. coli* towards gut compounds

To analyze the intracellular response of the chemotaxis pathway of *E. coli* towards gut compounds, we used a previously described assay based on Förster (fluorescence) Resonance Energy Transfer (FRET). Here, the phosphorylation-dependent interaction between CheY fused to yellow fluorescent protein (CheY-YFP) and its phosphatase CheZ fused to cyan fluorescent protein (CheZ-CFP) is monitored as a readout of the pathway activity [43, 44] (Fig. S1). The response was tested by stimulating cells expressing the FRET pair with serial dilutions of chemicals and measuring the subsequent change in FRET ratio (i.e., the ratio of the YFP to CFP fluorescence emission). As previously shown [43, 45, 46], stimulation with an attractant results in a rapid decrease in the FRET ratio, reflecting a decrease in the kinase activity. Stimulation with a repellent has an opposite effect, i.e., it increases the FRET ratio. Because continuous stimulation elicits adaptive changes in receptor methylation that gradually offset the effects of either attractant or repellent, the FRET ratio typically transiently overshoots upon removal of the chemoeffector (Fig. S1) [43, 44].

**Fig. 1:**
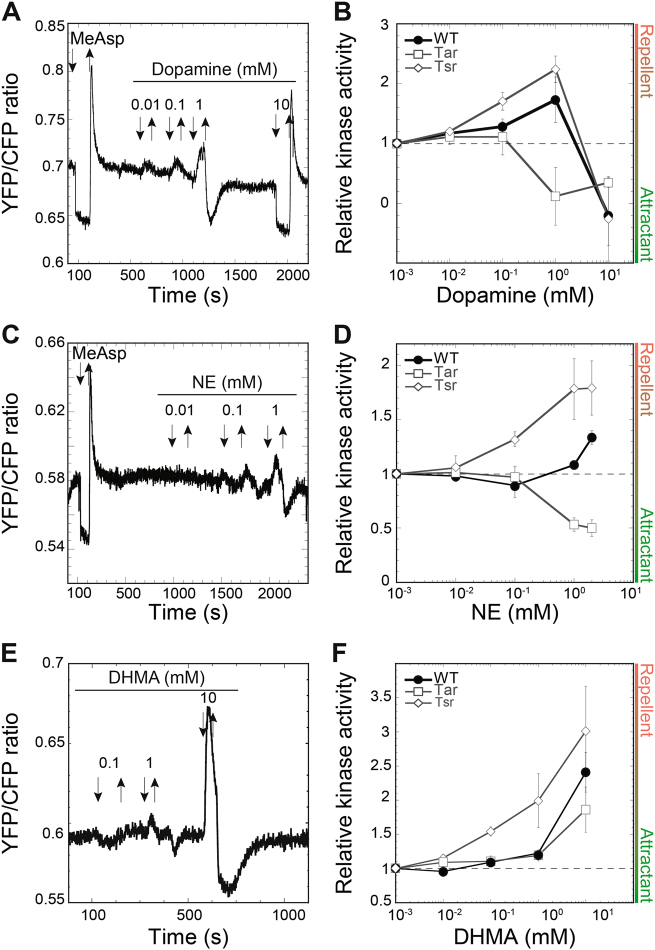
FRET-based analysis of the chemotaxis pathway response to catecholamines. **a**, **c**, **e** Examples of FRET measurements of responses to dopamine (**a**), norepinephrine (NE) (**c**), and 3,4-dihydroxymandelic acid (DHMA) (**e**). The ratio of YFP/CFP fluorescence reflects the efficiency of FRET and thus activity of the chemotaxis pathway. Buffer-adapted cells were stimulated with step-like addition and subsequent removal of compounds, indicated by downward and upward arrows, respectively. Saturating stimulation with 1 mM α-methyl-DL-aspartate (MeAsp) was used as a positive control. **b**, **d**, **f** Dose-response curves of wild-type cells (filled circles), Tar-only cells (open squares) or Tsr-only cells (open diamonds) to dopamine (**b**), NE (**d**), and DHMA (**f**). Each point represents the mean FRET-measured values of the kinase activity, normalized to the activity in buffer, from at least three independent experiments, with error bars indicating the standard error of the mean. Values above one correspond to a repellent response, while values below one correspond to an attractant response

We started our analysis with the biosynthetic pathway for catecholamine hormones (Fig. S2), the most widely studied compounds in molecular endocrinology. In the wild-type *E. coli* cells, the two major neurotransmitters of the catecholamine pathway, dopamine and NE, elicited biphasic responses (Fig. 1a–d). Dopamine was sensed as a repellent at concentrations below 1 mM, with the FRET ratio increasing upon addition of dopamine, and then decreasing upon its removal, which is opposite to the effect of canonical attractant α-amethyl-d,l-aspartate (MeAsp) (Fig. 1a). In contrast, at 10 mM dopamine signaled as attractant (Fig. 1a, b).

The response to NE was generally less pronounced, and it had an inverse pattern compared to the dopamine response. NE behaved as a weak attractant at low concentrations, but it produced a repellent response above 1 mM (Fig. 1c, d). The attractant response to low concentrations of NE is overall consistent with previous microfluidic measurements, although in those studies the chemotaxis towards low levels of NE was stronger and comparable to the chemotaxis towards amino acid attractants [39, 40]. This response was shown to rely on the conversion of NE into DHMA, which could be induced by the NE-mediated signaling [40–42]. Indeed, we observed that the responses of the wild-type *E. coli* cells to NE and DHMA were similar, with DHMA eliciting a weak attractant response up to 50 µM but a strong repellent response at higher concentrations (Fig. 1e, f), but in contrast to those previous studies no further enhancement of response could be observed upon growing *E. coli* cultures in presence of NE (not shown). No effects on the chemotaxis pathway activity could be observed for l-tyrosine, consistent with previous work [28], or for L-3,4-dihydroxyphenylanine (L-DOPA) (Fig. S3A). Notably, although L-DOPA and l-tyrosine can be detected in the GI tract, these precursors are rapidly converted into dopamine in the gut lumen [8, 47–49] and therefore unlikely to form stable gradients. Finally, although epinephrine elicited an apparent repellent response (not shown), interpretation of these data was complicated by a strong autofluorescence of epinephrine that interfered with FRET measurements.

**Fig. 2:**
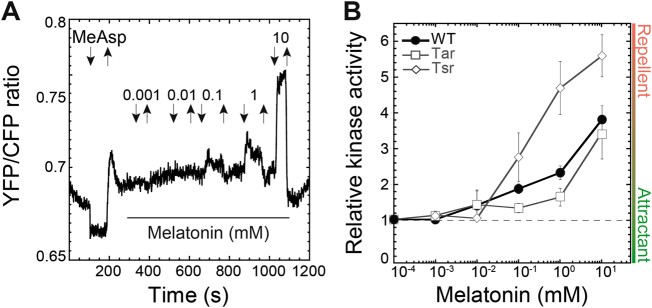
Pathway response to melatonin. **a** Example of a FRET measurement response in the wild-type cells, performed as in Fig. 1. **b** Dose–response curves from indicated strains. Error bars correspond to the standard error of the mean for three independent experiments

We next tested two major thyroid hormones, serotonin and melatonin. *E. coli* shows no chemotactic response to l-tryptophan, the precursor of thyroid hormones [28], and no obvious chemotaxis was observed for serotonin (Fig. S3B). However, the wild-type *E. coli* cells exhibited strong dose-dependent repellent response to melatonin at concentrations above 0.1 mM (Fig. 2a, b).

**Fig. 3:**
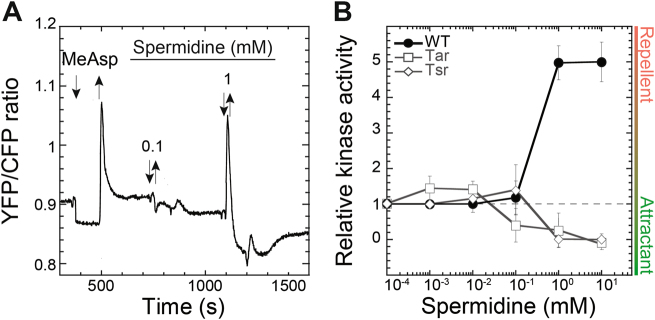
Pathway response to spermidine. **a** Example of FRET measurement of the chemotaxis pathway response in the wild-type cells, performed as in Fig. 1. **b** Dose–response curves from indicated strains. Error bars correspond to the standard error of the mean of three independent experiments

Finally, of the two tested polyamines, *E. coli* displayed no significant reaction to putrescine (Fig. S3B) but showed a strong repellent response towards spermidine in the millimolar concentration range (Fig. 3a, b).

### Responses mediated by Tar and Tsr

To further characterize the roles of the two most abundant *E. coli* chemoreceptors—Tar and Tsr—FRET measurements were also performed in strains expressing only one of them at levels similar to the net endogenous chemoreceptor expression in the wild type. We observed that both Tar and Tsr could mediate—sometimes opposite—responses to most of the tested hormones, indicating that chemotaxis of wild-type *E. coli* results from interplay between Tar- and Tsr-mediated responses. The Tsr-mediated response apparently makes a larger contribution to the behavior of the wild-type cells, consistent with Tsr being the most abundant receptor under our growth conditions [50, 51]. Specifically, Tar mediated an attractant response to dopamine (Fig. 1b and Fig. S4A), whereas the Tsr-only strain showed the same biphasic trend as the wild type, switching from repellent to attractant (Fig. 1b and Fig. S4B). For NE, Tar showed an attractant response, whereas Tsr sensed NE as a repellent over the entire concentration range (Fig. 1d and Fig. S4C,D). For DHMA, both Tar and Tsr mediated repellent responses (Fig. 1f and Fig. S4E, F). These results suggest that the NE sensing at high concentrations could not be solely explained by its conversion into DHMA, since Tar responses to NE and DHMA were clearly different. Both Tar and Tsr mediated a repellent response to melatonin, similar to the one observed for the wild-type strain (Fig. 2b and Fig. S4G, H).

In contrast to the observed responses to hormones, the wild type sensing of spermidine could clearly not be accounted by a combination of Tar- and Tsr-mediated responses (Fig. 3b). These were weak and biphasic, with attractant responses at high concentration where the wild type showed a stronger repellent response, indicating that spermidine sensing in the wild type *E. coli* must be mediated by one of the minor receptors.

### Microfluidic assay of chemotaxis

**Fig. 4:**
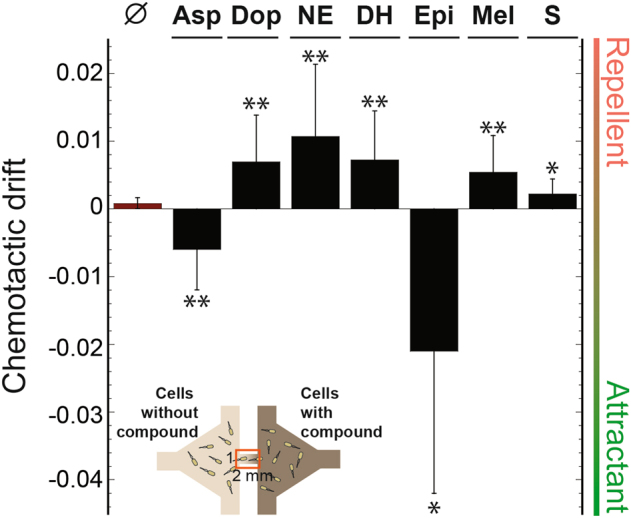
Chemotactic response in gradients of gut compounds. Chemotactic drift (defined as *v*_*ch*_/*αv*_*0*_, see Methods) was measured in gradients of dopamine (Dop), NE, DHMA (DH), epinephrine (Epi), melatonin (Mel) or spermidine (S) established in a microfluidic device (Inset; measurement area is indicated by an orange rectangle). Zero chemotactic drift corresponds to non-responding cells and one to direct swimming up (negative values) or down (positive values) the gradient. For the negative control (∅), drift of the wild-type cells was measured in buffer in the absence of gradients. For the positive control, the drift was measured in a gradient of 0–1 mM aspartate (Asp). As a reference (not shown), the chemotactic drift of the wild-type cells in a gradient of non-metabolized attractant MeAsp had an average value of −0.1 [52]. Error bars indicate the standard error of the mean. A one-tailed student *t*-test was performed to assess the significance of the response being different from 0 (**P ≤ 0.05; *P *≤* 0.1)

To verify the chemotactic responses to the gut compounds measured by FRET, we additionally analyzed the chemotactic behavior of *E. coli* in microfluidic channels. Here we used a recently described assay that allows measurements of the average motion of a bacterial population in linear chemical gradients, characteristic for chemotaxis [52, 53]. The observed chemotactic behavior (Fig. 4 and Fig. S5) was consistent with FRET measurements. The wild-type strain showed a statistically significant repellent taxis to all tested hormones in the 0–1 mM gradient, similar to the dominant response observed in this concentration range by FRET, except for epinephrine, which attractant taxis that is consistent with a previous report [39] was observed (Fig. 4). Chemotaxis of *E. coli* cells expressing only Tar or Tsr was also generally consistent with the FRET data, although in several cases the responses were variable and may require further verification (Fig. S5). Notably, only the wild type showed strong repellent reaction to spermidine, whereas the Tar- and Tsr-only strains showed either attractant or no response. Although the observed chemotactic drift away or towards the gut compounds was overall weaker than the drift in gradients of MeAsp [52, 53], it was similar to chemotaxis observed toward the metabolized strong attractant aspartate (Fig. 4). This suggests that degradation or uptake of attractants can markedly weaken the response that can be measured using this microfluidic assay, which may not be surprising given the relatively high density of bacteria within the channel in these experiments.

### Mechanism of the spermidine response

**Fig. 5:**
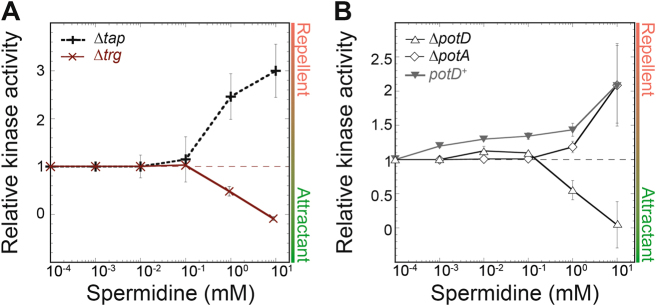
Dose-response curves of the pathway response to spermidine. Responses were measured by FRET as in Fig. 1 for **a** ∆*trg* cells (red crosses) and ∆*tap* cells (black crosses) and **b** *∆potD* (open triangles), ∆*potA* (open diamonds), and ∆*potD/potD*^+^ (filled inverted triangles) cells (see Figure S6). Differences between Δ*trg* and Δ*tap* responses in **a** and between Δ*potD* and Δ*potA* or ∆*potD/potD*^*+*^ responses **b** are significant according to student *t*-test performed with responses to 10 mM spermidine (*P* **≤** 0.01)

As mentioned above, our data indicated that the strong repellent response to spermidine observed in the wild type is likely to be mediated by one of the low-abundance receptors, Tap or Trg. As neither Trg nor Tap can function as the only receptor in *E. coli* [54], we tested chemotaxis to spermidine with *trg* and *tap* deletion strains (Fig. 5a). Whereas *∆tap* cells responded to spermidine like the wild type, the response of *∆trg* cells was comparable to the one observed for the Tar- and Tsr-only strains (Fig. S6A). This clearly suggests that the repellent response to spermidine is mediated by Trg. To further confirm the involvement of Trg, wild-type cells were adapted to ribose (a Trg-specific attractant) before stimulation with spermidine (Fig. S6B). Adaptation to ribose indeed abolished the repellent response to spermidine, implying that the interaction of the periplasmic ribose-binding protein (RBP) with the sensory domain of Trg, which is known to mediate response to ribose [55, 56], interferes with spermidine sensing.

Signaling via periplasmic binding proteins (BPs) is common not only to ribose but to all Trg- or Tap-specific chemoattractants for which the sensing mechanisms have been established. These periplasmic BPs are components of the ATP-binding cassette (ABC) transporters, but they also interact with the low-abundance receptors and regulate their activity upon binding to ligands. We thus hypothesized that the preferential *E. coli* ABC transporter for spermidine, PotABCD [24, 57], might be involved in the Trg-mediated response. In the PotABCD transporter complex, PotD is the periplasmic BP, PotA is the membrane-associated ATPase, and PotB and PotC are two membrane-spanning components of the transmembrane channel [24, 58]. Notably, the crystal structure of the PotD protein complex with spermidine is very similar to *E. coli* d-Glucose/d-Galactose-binding protein (GBP) [59], another interaction partner of Trg.

Indeed, in the strain deleted for *potD*, the specific repellent response to spermidine was abolished, similar to the effect of *trg* deletion (Fig. 5b and Fig. S6C). Complementation with ectopically expressed *potD* restored this response (Fig. S6D). In contrast, a *potA* strain deleted for the membrane-associated ATPase behaved similarly to the wild type (Fig. 5b). Hence, our data suggest that the interaction of the periplasmic BP PotD with Trg—and not spermidine uptake—is required for the Trg-mediated repellent response to spermidine.

### Effects of the gut compounds on *E. coli* growth

**Fig. 6:**
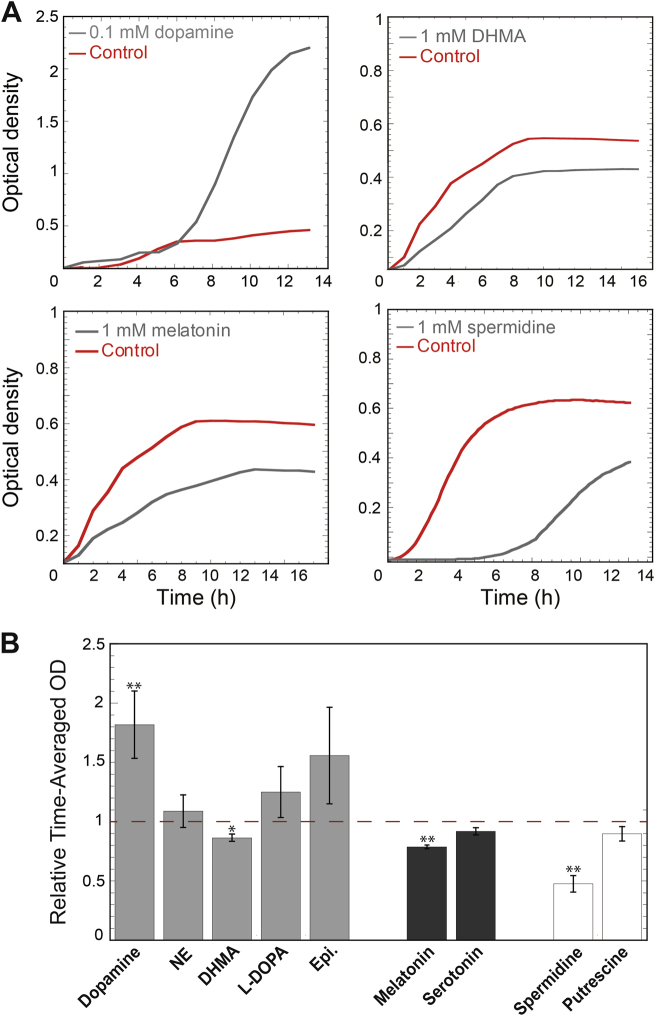
Effect of the gut compounds on *E. coli* growth. **a** Examples of growth curves of *E. coli* MG1655 grown at 37 °C in TB containing dopamine, DHMA, melatonin or spermidine at indicated final concentrations (black line), and of control culture grown in TB (red line). Optical density of culture was measured at 600 nm as described in Methods. **b** The time-averaged OD, calculated as the area below the growth curves and divided by the duration of the experiment. For each experiment, the time-averaged OD was normalized to the time-averaged OD of the control culture in TB. Gray bars represent the catecholamine group; black bars the thyroid group; and white bars the polyamine group. A one-tailed student *t*-test was performed to assess the significance of the difference from the control (**P ≤ 0.01; *P ≤ 0.05). Each bar represents the mean of at least three independent experiments, with error bars indicating standard deviation

To better understand the physiological relevance of the observed chemotactic responses, we analyzed the effects of tested compounds on growth of a planktonic *E. coli* culture. Indeed, several compounds affected different stages of *E. coli* growth (Fig. 6a, Fig. S7). To capture these effects with a single number, we calculated the area below the curve as a measure of time-averaged OD (Fig. 6b). Similar differences between cultures were observed when comparing their maximal OD (Fig. S8). We observed that L-DOPA, epinephrine and, particularly, dopamine enhanced *E. coli* growth, in agreement with previous reports [11–13]. As oxidation of dopamine results in the development of black color during incubation, which could lower the precision of the optical density measurements despite baseline subtraction, we directly confirmed the growth-promoting effect of dopamine by quantifying the number of viable cells in the culture (Fig. S9). Although the exact mechanism of this growth stimulation by dopamine remains to be investigated, it might be related to the dopamine-mediated enhancement of iron uptake [11, 12].

In contrast, DHMA and melatonin were growth-inhibitory, and the strongest growth inhibition was observed for spermidine (Fig. 6a, b and Fig. S8). The effect of spermidine was only observed above 1 mM, which matches the concentration range of chemorepellent response. In summary, the chemotactic preferences of *E. coli* were seemingly consistent with the effects of the tested compounds on *E. coli* growth and with their expected stability in the GI tract (Table S1).

## Discussion

While most of the microbial endocrinology studies have focused on the effects of bacterial species on the concentrations of hormones in the GI tract, less attention has been paid to how the microbes themselves recognize these compounds [4–7, 60]. In this study, we used *E. coli* as the model enteric bacterium to investigate chemotactic responses to a range of compounds that are present in the human gut. Remarkably, we observed chemotactic responses to compounds that are known to accumulate in the gut—with the notable exception of serotonin—namely dopamine, NE, epinephrine, DHMA, melatonin and spermidine [8, 61–64]. Although accurate concentration measurements across the gut are complicated by the heterogeneity of the gut environment and the strong dependence on food content and health state of the host [65–67], these compounds can reach micro- to millimolar levels across the gut lumen [8, 10, 68]. Even higher concentrations are expected to be present in the vicinity of the mucous layer where these compounds are secreted, consistent with the observed concentration range of the chemotactic response. In contrast, no response could be detected for their precursors with similar chemical structure (e.g., l-tyrosine, l-tryptophan, l-DOPA, and putrescine), which are primarily derived from food sources and rapidly turned over in the GI tract [10, 48]. They are therefore unlikely to form long-lived gradients that can be used for orientation in the intestine. Our results thus provide further evidence for the hypothesis that bacteria can specifically utilize host signals to detect their GI location [14, 39–42].

For all hormones analyzed, chemotaxis in the wild type appears to be the result of interplay between responses mediated by the two high-abundance *E. coli* chemoreceptors, Tar and Tsr. For the most part, the Tsr-mediated response makes a larger contribution, in agreement with the higher expression of Tsr under our growth conditions. Such interplay between Tar- and Tsr-mediated responses is similar to the previously characterized tactic behavior in gradients of pH and temperature [69, 70], but contrasts with the responses to conventional chemoattractants that specifically bind to the periplasmic sensory domains of the receptors. Considering the similar range of hormone concentrations that are detected by Tar and Tsr, it is likely that all tested hormones are sensed indirectly, by perturbing some aspect of cell physiology. Although the exact molecular mechanisms of this sensing remains to be elucidated, it nevertheless appears to be specific, since chemically closely related precursor compounds elicit no response.

Furthermore, there seemed to be a correlation between the chemotactic preferences and effects of individual compounds on *E. coli* growth (Table S1). Although this relation was less clear for hormones that elicited biphasic responses—dopamine and NE—high concentrations of dopamine led to a strong attractant response that was consistent with the growth-promoting effect of dopamine. NE had no significant effect on growth, but since NE may play a role in the activation of the immune response [6, 71], its sensing may be important for avoidance of the immune system and thus for bacterial survival [6, 13, 72].

For the compounds that elicited a repellent response at high concentrations, namely DHMA, melatonin and spermidine, there was a pronounced correlation with growth inhibition. Although low concentrations of DHMA were previously shown to elicit a highly sensitive attractant response [40–42], at high concentrations of DHMA that affect growth, the behavior of *E. coli* in gradients is apparently dominated by the repellent response. The chemotactic response to melatonin and the observed growth inhibition are consistent with recently proposed effects of this hormone on the microbiome [17, 73]. In the context of the correlation observed between chemotaxis and growth effects, it is interesting that serotonin affects neither growth nor chemotaxis of *E. coli*.

Of the two tested polyamines, putrescine and spermidine, only the latter elicited a chemotactic response. This might be consistent with putrescine being rapidly taken up or converted to spermidine or spermine in the small intestine [10]. In contrast, levels of spermidine in the GI tract are expected to be high [10, 74]. The only compound that elicited a straight attractant response in the wild-type cells was epinephrine, consistent with a previous study [39] and correlating again with its growth-promoting effect for *E. coli*.

Although the mechanism of hormone sensing by *E. coli* chemoreceptors remains to be investigated, we could show that spermidine is specifically sensed as a repellent by the low abundance receptor Trg. So far, Trg has been only implicated in attractant responses to ribose, glucose and galactose, mediated by the interactions of its sensory domain with periplasmic binding proteins, RBP (for ribose) and GBP (for glucose and galactose). Our results suggest that the response to spermidine similarly involves the periplasmic binding protein PotD, which is part of the spermidine uptake system PotABCD. To our knowledge, this is the first example of a repellent response mediated by a minor chemoreceptor. It is also the first clear example of a repellent response involving a periplasmic binding protein. Although nickel-binding protein had been implicated initially in the repellent response to nickel mediated by Tar [75], this finding was subsequently disproved [76].

In general, considering the role of chemotaxis in the host–microbe interactions may provide a deeper understanding of the behavior of enteric bacteria within the host. Because bacterial chemotaxis is primarily a single-cell behavior, chemotaxis may be highly important for the survival and proliferation of *Proteobacteria* in the GI tract [38], even though they represent only minor constituents of the normal gut microbiota. The apparent correlation found between chemotactic preferences, growth effects and the nature of tested gut compounds (i.e., food-derived or secreted) suggest that bacteria are exposed to gradients of hormones and other host-derived compounds in the mammalian gut. Consequently, *E. coli*, and most likely other motile enteric bacteria, seem to have evolved specific tactic responses as a way not only to avoid harmful (or to locate beneficial) levels of these compounds, but also to orient themselves in the gut. In this context, repellent responses observed at high concentrations of compounds that are secreted from the gut epithelium may enable bacteria to limit immediate contact with the mucous layer of the GI tract, which is known to contain high levels of antimicrobial proteins and immunoglobulin A (IgA) [1, 77]. Furthermore, the interplay between these repellent responses and attractant responses mediated by low concentrations of NE and DHMA, as observed previously [14, 39–42] and in our work, could explain chemotactic accumulation of *E. coli* at a certain distance from the mucosal surface [78]. This area might represent a specific growth niche in the intestine [79], where bacteria can benefit from rapidly diffusing nutrients released by the epithelium without being harmed by the mucosal antimicrobials. Intestinal inflammation has been previously shown to lead to an enhance release of nutrients [38] and electron acceptors [36, 80] along with a reduced hormone secretion [81–83]. This might shift the accumulation pattern of EHEC or *Salmonella*, which possess chemotaxis systems that are nearly identical to that of commensal *E. coli*, towards the mucosal surface, possibly promoting proliferation of *Proteobacteria* and infection [84].

## Methods

### Bacterial strains and plasmids

All strains and plasmids used in this work are listed in Table S2. Strains used for the microfluidics and FRET measurements were derived from *E. coli* RP437 [85], the wild-type strain for chemotaxis. FRET strains were transformed with a plasmid expressing CheY-YFP and CheZ-CFP pair from a bi-cistronic construct pVS88 [43, 44], and where indicated with a plasmid expressing either Tar (pVS1092) or Tsr (pVS160). Strain VS104 [Δ(*cheY cheZ*)] carrying pVS88 was used as the wild-type for FRET. The ∆*potD* and ∆*potA* deletions were introduced by phage P1_vir_ transduction from the respective mutants from the Keio collection [86]. Km^R^ cassettes were eliminated via FLP recombination [87]. For *potD* complementation (*potD*^*+*^), ∆*potD* was transformed with the constructed plasmid derivative of pKG116 containing *potD* (pJL02). For growth experiments, MG1655 was used as *E. coli* wild type.

### Reagents

l-Tyrosine (≥98% purity), L-Dopa-(phenyl-d3) (L-3,4-dihydroxyphenylalanine, 98% purity), Dopamine hydrochloride, (−)-Norepinephrine,(−)-Epinephrine (≥98% purity), DL-3,4-Dihydroxymandelic acid (DHMA, 98% purity), Serotonin hydrochloride, Melatonin powder (≥98% purity), Putrescine dihydrochloride, Spermidine (≥99% purity), α-methyl-DL-aspartate (MeAsp), were obtained from Sigma-Aldrich (Taufkirchen, Germany), and l-serine from Acros Organics—Thermo Fisher Scientific (Nidderau, Germany).

### FRET assay

The FRET measurements were performed as previously described [43, 46]. Briefly, *E. coli* cells were grown in tryptone broth (TB) media (1% tryptone, 0.5% NaCl), supplemented with the respective antibiotics (100 mg/mL ampicillin; 17 mg /mL chloramphenicol) and inducers (Table S2) at 34 °C and 275 r.p.m. Cells were harvested at OD_600_ 0.6. by centrifugation (4000 × *g* for 5 min), washed with tethering buffer (10 mM KPO_4_, 0.1 mM EDTA, 1 μM methionine, 10 mM lactic acid, pH 7), and stored at 4 °C for 30 min. The sample was attached to a polylysine-coated coverslip, placed in a flow chamber under constant flow (300 µl/min) of tethering buffer using a syringe pump (Harvard Apparatus, Massachusetts, United States), which was used for stimulation with compounds of interest (Fig. S1). Measurements were performed on an upright fluorescence microscope (custom-modified Zeiss Axiovert 200 microscope, Carl Zeiss Microscopy GmbH, Jena, Germany) equipped with a PCI-6034 counting board connected to a computer with custom written LabView7 software (National Instruments). CFP fluorescence was excited at 436/20 nm through a 455 nm dichroic mirror by a 75 W Xenon lamp. To detect CFP and YFP emissions, 480/40 nm band pass and 520 nm long pass emission filters were used, respectively. Fluorescence of a monolayer of 300–500 cells was continuously recorded in the cyan and yellow channels using photon counters with a 1.0 s integration time. FRET response was measured as the change in the ratio of YFP/CFP due to energy transfer and normalized to the response of buffer-adapted cells to saturating stimulation with known strong attractant, either α-methyl-d,l-aspartate (MeAsp) or l-serine.

### Microfluidics assay

The measurement of the chemotactic drift was performed as previously described [52]. Briefly, *E. coli* RP437 cells grown to mid-exponential growth phase were harvested by centrifugation (4000 × *g* for 5 min), washed with chemotaxis buffer (10 mM KPO_4_, 0.1 mM EDTA, 67 mM NaCl, pH 7) and stored at 4 °C for 30 min to inhibit protein synthesis. The sample was placed in silicone elastomer (SYLGARD 184, 1:10 crosslinker to base ratio, Dow Corning, USA) hand-made chemotaxis chamber, consisting of two reservoirs linked via a small channel (length L = 2 mm, width w = 1 mm). One chamber contains suspended bacteria and the other contains suspended bacteria mixed with the target compound at the indicated concentration. After 30 min, a linear gradient of chemoattractant is formed in the channel to which the cells respond. The response of each strain to the gradient was recorded in the middle of the channel using video-microscopy (×10 magnification under phase contrast illumination, Mikrotron 4CXP camera running at 100 frames per seconds for 100 s, with a 717 × 717 μm^2^–512 × 512 px2 –field of view, focal plane halfway through the 50 μm sample depth). A high-throughput computer analysis of the films yielded the average chemotactic drift velocity of the population *v*_*ch*_, the population-averaged swimming speed of the cells *v*_*0*_ and the fraction of swimming cells, *α*, which enables to estimate the chemotactic drift *v*_*ch*_/*αv*_0_, where zero corresponds to non-responding cells and one to a population where all cells swim directly down the gradient.

### Growth experiments

*E. coli* cells from an overnight culture (37 °C and 200 r.p.m. in TB) were diluted until OD_600_ 0.05 in a total volume of 110 µl in a 96-well plate (Greiner Bio-One, Frickenhausen, Germany). OD_600_ was measured using a plate reader (Tecan Infinite M1000, Tecan Deutschland GmbH, Crailsheim, Germany) for 14 h at 37 °C and 180 r.p.m. TB medium was supplemented with the analyzed compounds at the indicated concentrations. For the dopamine experiments, an additional baseline subtraction was performed, with TB without cells but supplemented with dopamine as a negative control. Growth was analyzed by calculating the area under the curve divided by the duration for each individual experiment (14 h), giving the time-averaged OD. The average of all experiments was then calculated and normalized to the control. Statistical analysis was performed to assess the difference to the control with a one-tailed student *t*-test. *P*-values lower than 0.05 were considered statistically significant.

### Colony forming unit (CFU) assay

The CFU assay was performed by diluting an overnight culture 1:100 in a total volume of 10 ml of TB containing dopamine at indicated concentrations and growing it at 37 °C for 6 h. Cells were serially diluted 10^−6^ and 10^−7^ and 100 µl of the samples were plated on LB plates in triplicates and incubated at 37 °C over night. The number of colonies in each plate was counted and the CFU/ml (number of colonies/volume of inoculation×dilution) was calculated.

## Electronic supplementary material

Figure S1

Figure S2

Figure S3

Figure S4

Figure S5

Figure S6

Figure S7

Figure S8

Figure S9

Table S1

Table S2
