# Supplementary material for: Chemotaxis of Escherichia coli to major hormones and polyamines present in human gut
Source: ISME J. 2018 Jul 11;12(11):2736–47. doi: 10.1038/s41396-018-0227-5 (PMC6194112; doi:10.1038/s41396-018-0227-5)
Supplement: Supplementary file 1 — Figure S1 [file 41396_2018_227_MOESM1_ESM.pdf]

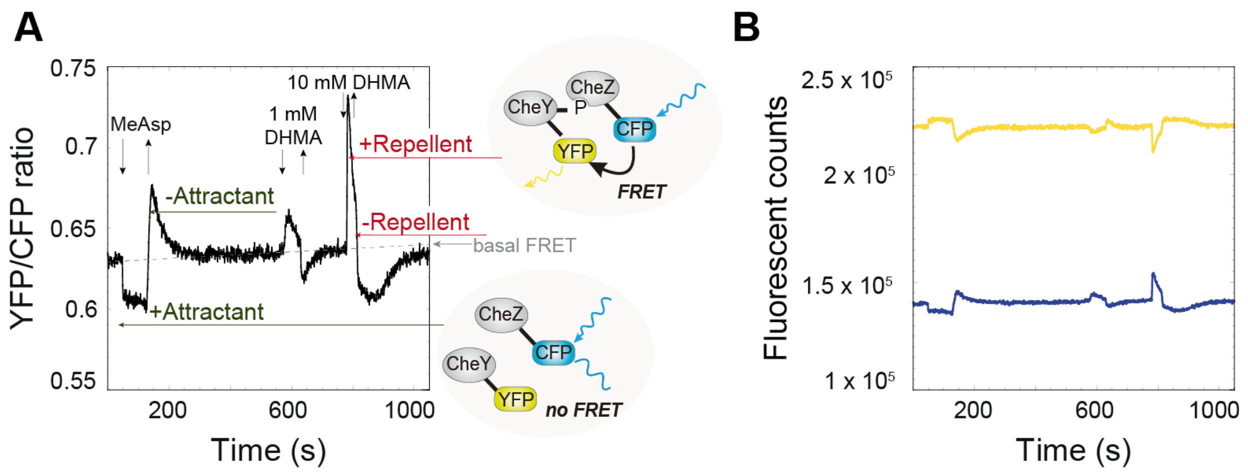

Figure S1: FRET-based analysis of *E. coli* chemotaxis pathway responses. (A) Wild-type cells expressing CheY-YFP and CheZ-CFP were stimulated by addition and subsequent removal of attractant, 1 mM α-methyl-DL-aspartate (MeAsp), and by addition and subsequent removal of repellent, 1 mM and 10 mM 3,4-dihydroxymandelic acid (DHMA), at the time points indicated by arrows. Attractants inhibit the kinase activity of CheA, leading to a decrease in FRET, which is observed as a decrease in the YFP/CFP ratio due to the reduced numbers of CheY-P-CheZ complexes. Conversely, removal of attractants or stimulation by repellents leads to an increase in the YFP/CFP ratio. **(B)** Time traces of fluorescence intensity in the YFP (yellow) and CFP (cyan) channels for the FRET response observed in (A). Opposite changes in the two channels characterize specific FRET response.
