# Supplementary figures and images for: Chemotaxis of Escherichia coli to major hormones and polyamines present in human gut
Source: ISME J. 2018 Jul 11;12(11):2736–47. doi: 10.1038/s41396-018-0227-5 (PMC6194112; doi:10.1038/s41396-018-0227-5)

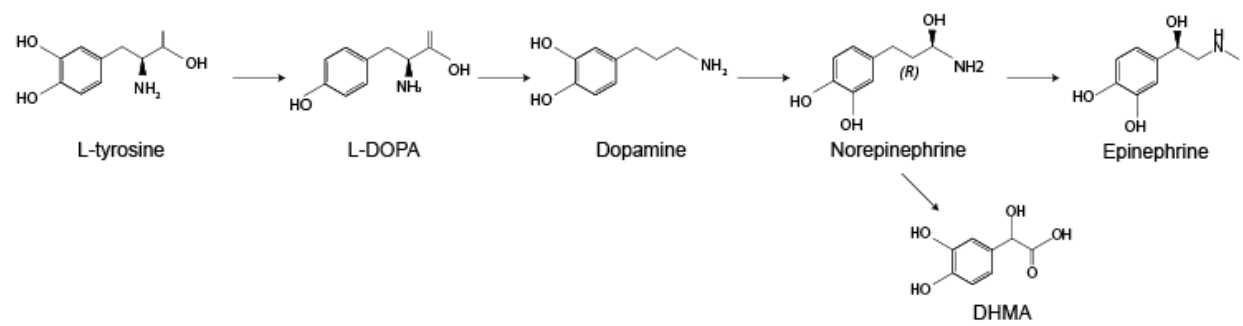

**Figure S2: Biosynthetic pathway of catecholamines.**

Supplement: Supplementary file 2 — Figure S2 [file 41396_2018_227_MOESM2_ESM.pdf]
