# Supplementary material for: Chemotaxis of Escherichia coli to major hormones and polyamines present in human gut
Source: ISME J. 2018 Jul 11;12(11):2736–47. doi: 10.1038/s41396-018-0227-5 (PMC6194112; doi:10.1038/s41396-018-0227-5)
Supplement: Supplementary file 3 — Figure S3 [file 41396_2018_227_MOESM3_ESM.pdf]

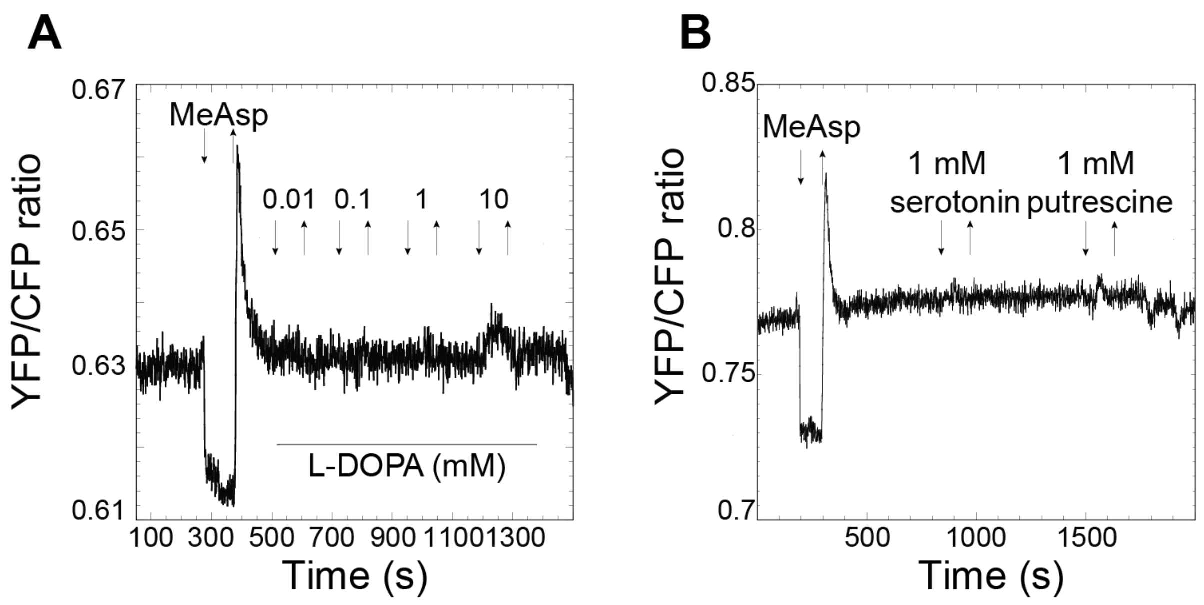

Figure S3: FRET measurements of the wild-type response to L-3,4-dihydroxyphenylanine (L-DOPA) **(A)** and serotonin and putrescine **(B).** Measurements were performed and plotted as in Figure 1.
