# Supplementary material for: Chemotaxis of Escherichia coli to major hormones and polyamines present in human gut
Source: ISME J. 2018 Jul 11;12(11):2736–47. doi: 10.1038/s41396-018-0227-5 (PMC6194112; doi:10.1038/s41396-018-0227-5)
Supplement: Supplementary file 4 — Figure S4 [file 41396_2018_227_MOESM4_ESM.pdf]

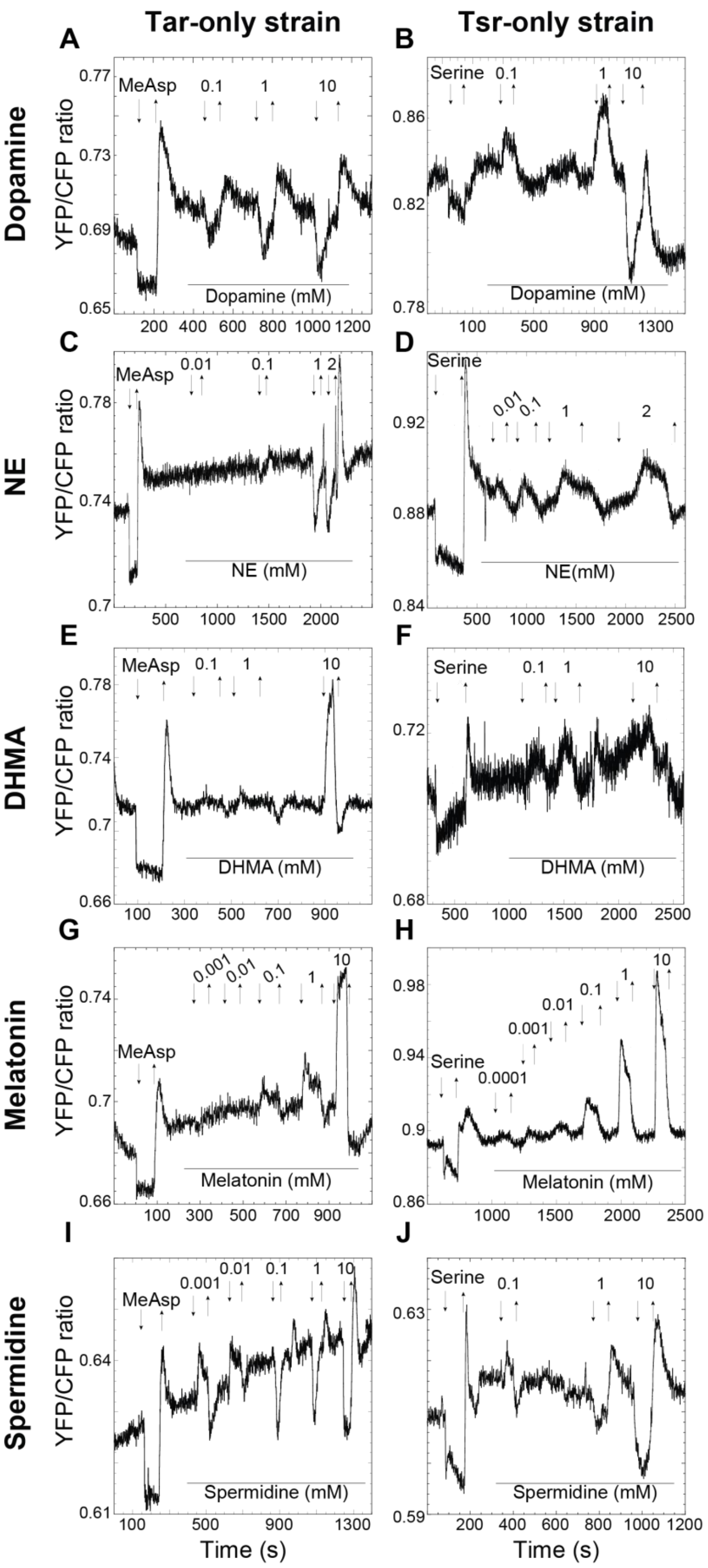

Tar-only strain
Tsr-only strain
A
B
C
D
E
F
G
H
I
J
Dopamine
NE
DHMA
Melatonin
Spermidine
YFP/CFP ratio
MeAsp
Serine
Dopamine (mM)
NE (mM)
NE(mM)
DHMA (mM)
Melatonin (mM)
Spermidine (mM)
Time (s)

Figure S4: Examples of FRET measurements for Tar-only cells (left side) and Tsr-only cells (right side). Responses to dopamine **(A, B)**, NE **(C, D)**, DHMA **(E, F)**, melatonin **(G, H)** and spermidine **(I, J)** are shown. Measurements were performed and plotted as in Figure1.
