# Supplementary material for: Chemotaxis of Escherichia coli to major hormones and polyamines present in human gut
Source: ISME J. 2018 Jul 11;12(11):2736–47. doi: 10.1038/s41396-018-0227-5 (PMC6194112; doi:10.1038/s41396-018-0227-5)
Supplement: Supplementary file 5 — Figure S5 [file 41396_2018_227_MOESM5_ESM.pdf]

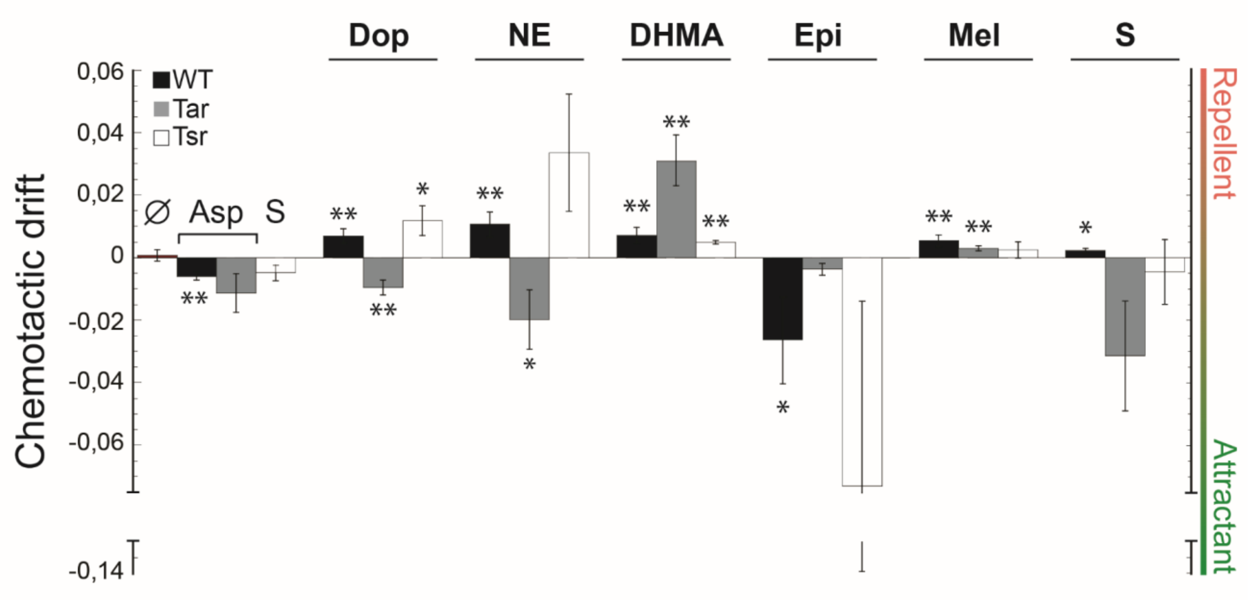


**Figure S5: Chemotactic response in gradients of gut compounds.** Chemotactic drift (defined as $v_{ch}/\alpha v_0$, see Methods) was measured in gradients of dopamine (Dop), NE, DHMA, epinephrine (Epi), melatonin (Mel) or spermidine (S) established in a microfluidic device. Measurements were performed and plotted as in Figure 4. For the negative control (Ø), drift of the wild-type cells was measured in buffer in absence of gradients. For the positive control, the drift was measured in a gradient of 0 to 1 mM aspartate (Asp) for the wild-type and Tar-only cells or in a gradient of 0 to 1 mM serine (S) for Tsr-only cells. Error bars indicate the standard error of the mean. A one-tailed student *t*-test was performed to assess the significance of the response being different from 0 (**: $P \leq 0.05$; *: $P \leq 0.1$).
