# Supplementary material for: Chemotaxis of Escherichia coli to major hormones and polyamines present in human gut
Source: ISME J. 2018 Jul 11;12(11):2736–47. doi: 10.1038/s41396-018-0227-5 (PMC6194112; doi:10.1038/s41396-018-0227-5)
Supplement: Supplementary file 6 — Figure S6 [file 41396_2018_227_MOESM6_ESM.pdf]

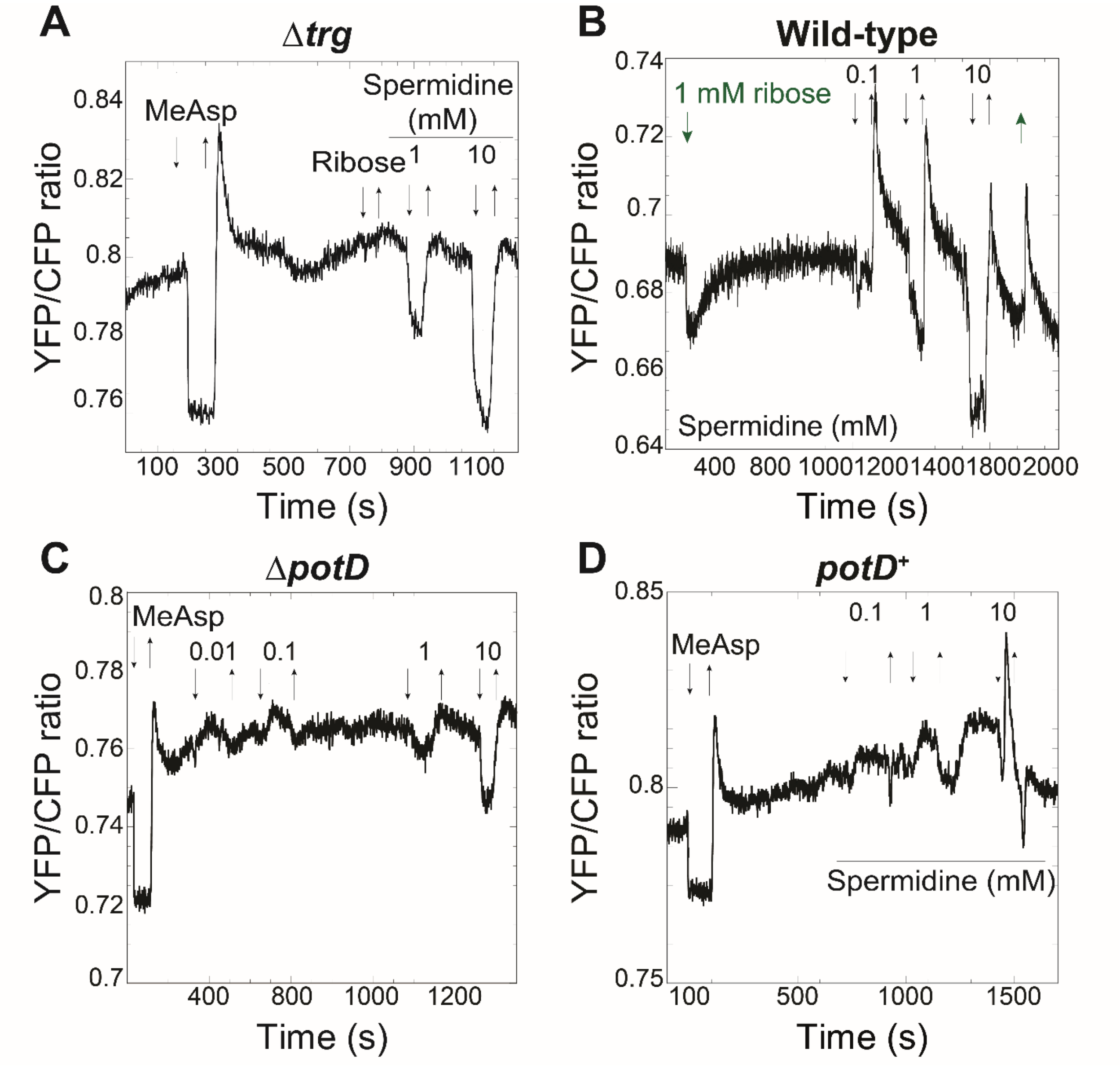

Figure S6: FRET measurements of responses to spermidine. Measurement example for Δ*trg* **(A)**, wild-type **(B)**, Δ*potD* **(C)** and Δ*potD* complemented with the plasmid pJL02 (*potD*$^{+}$) **(D)** strains, stimulated with indicated concentrations of spermidine. In (B) cells were adapted to 1 mM of ribose (indicated by the green arrows) and stimulated with spermidine in presence of ribose. Measurements were performed and plotted as in Figure 1.
