# Supplementary material for: Chemotaxis of Escherichia coli to major hormones and polyamines present in human gut
Source: ISME J. 2018 Jul 11;12(11):2736–47. doi: 10.1038/s41396-018-0227-5 (PMC6194112; doi:10.1038/s41396-018-0227-5)
Supplement: Supplementary file 7 — Figure S7 [file 41396_2018_227_MOESM7_ESM.pdf]

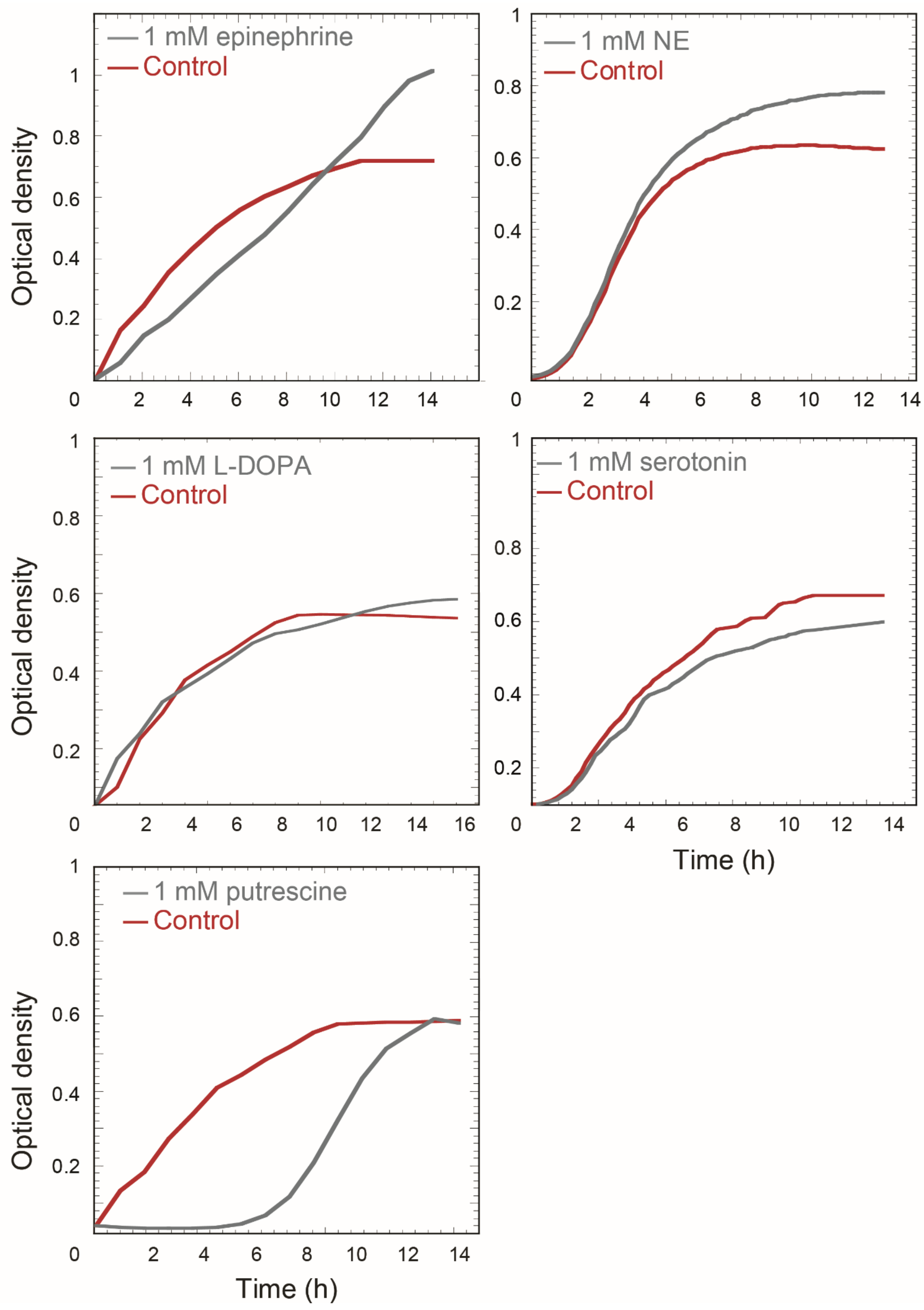

1 mM epinephrine
Control
1 mM NE
Control
1 mM L-DOPA
Control
1 mM serotonin
Control
1 mM putrescine
Control
Optical density
Time (h)

Figure S7: Example of growth curves in presence of gut compounds. MG1655 cells were grown as in Figure 6, at 37°C in TB (control – red curve) or TB supplemented with 1 mM of epinephrine, NE, L-DOPA, serotonin or putrescine as indicated (gray curves). The optical density (OD_{600}) was measured every hour.
