# Supplementary material for: Chemotaxis of Escherichia coli to major hormones and polyamines present in human gut
Source: ISME J. 2018 Jul 11;12(11):2736–47. doi: 10.1038/s41396-018-0227-5 (PMC6194112; doi:10.1038/s41396-018-0227-5)
Supplement: Supplementary file 8 — Figure S8 [file 41396_2018_227_MOESM8_ESM.pdf]

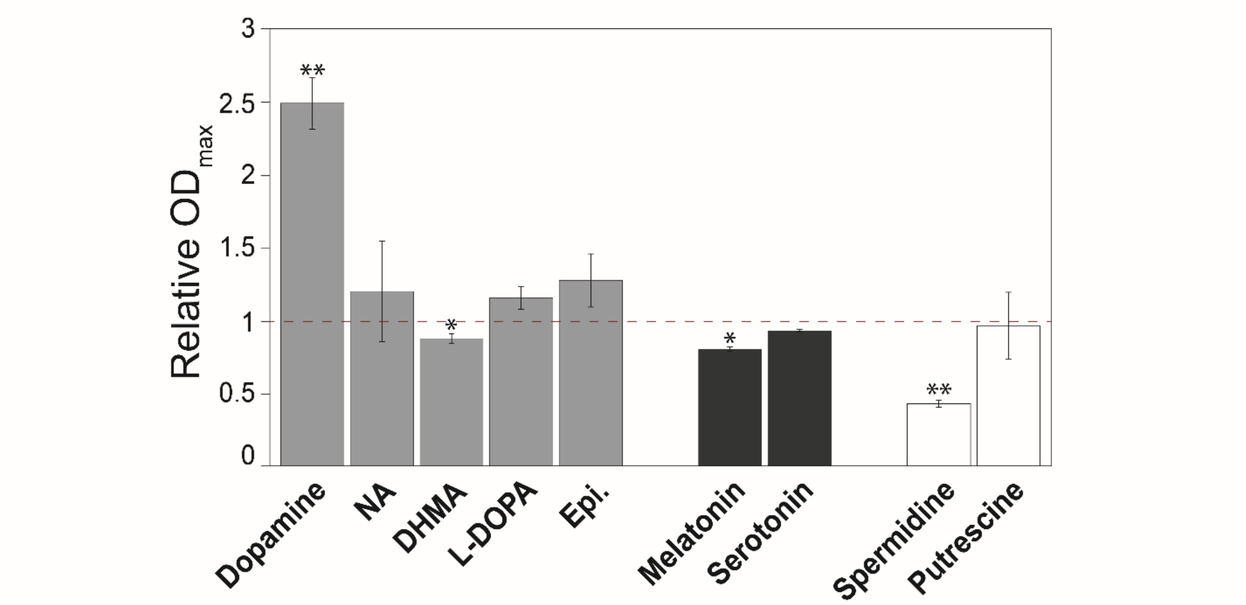

Figure S8: Effects of the gut compounds on maximal OD of *E. coli* culture. Cells were grown as in Figure 6 and culture density was determined after 14 h. OD_{600} values were plotted normalized to the control culture with untreated cells (dashed line). Error bars indicates the standard deviation.
