# Supplementary material for: Chemotaxis of Escherichia coli to major hormones and polyamines present in human gut
Source: ISME J. 2018 Jul 11;12(11):2736–47. doi: 10.1038/s41396-018-0227-5 (PMC6194112; doi:10.1038/s41396-018-0227-5)
Supplement: Supplementary file 9 — Figure S9 [file 41396_2018_227_MOESM9_ESM.pdf]

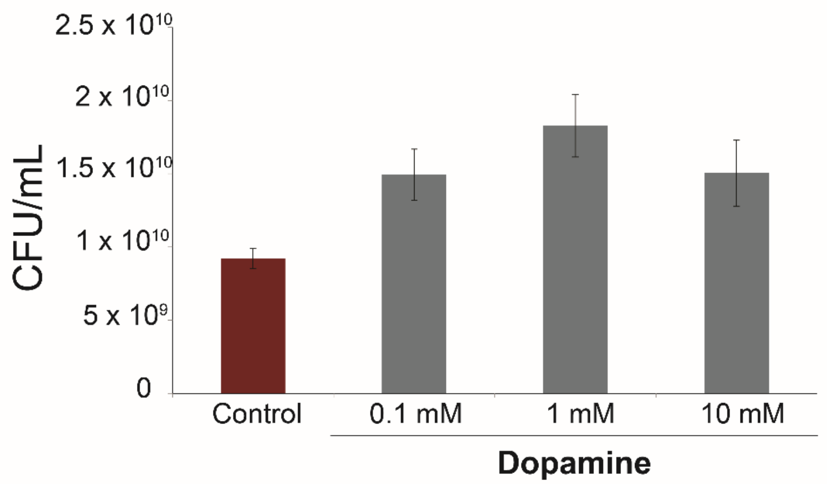

Figure S9: Effect of dopamine of the number of colony forming units (CFU) of *E. coli* culture. CFU count was determined on LB plates for the cultures grown in TB (control) and TB with indicated dopamine concentrations at 37°C, as described in Methods. Experiments were performed in triplicates. The CFU/ml is the number of colonies per volume of inoculation. Error bars indicate standard deviation.
