# Supplementary material for: Chemotaxis of Escherichia coli to major hormones and polyamines present in human gut
Source: ISME J. 2018 Jul 11;12(11):2736–47. doi: 10.1038/s41396-018-0227-5 (PMC6194112; doi:10.1038/s41396-018-0227-5)
Supplement: Supplementary file 10 — Table S1 [file 41396_2018_227_MOESM10_ESM.pdf]

Table S1: Effect of gut compounds on chemotaxis and growth of *E. coli*

Compounds	Chemotactic response[a]		Growth effect
	FRET	Microfluidics	
**L-tyrosine**	0	ND	0
**L-DOPA**	0	ND	0
**Dopamine**	-/+	-**	+***
**Norepinephrine**	+/-	-**	0
**Epinephrine**	ND	+*	+
**DHMA**	-	-**	-**
**Serotonin**	0	ND	0
**Melatonin**	-	-***	-***
**Putrescine**	0	ND	0
**Spermidine**	-	-*	-***

ND: not determined; 0: no response/effect; +: attractant/enhancement; -: repellent/inhibition

[a]Response of the wild-type cells.

*, **, *** One-tailed student t-test statistical significance to the control of $P \leq 0.1$, $P \leq 0.05$ and $P \leq 0.01$, respectively (based on Fig. 4 and Fig. 6B)
