# Supplementary material for: Chemotaxis of Escherichia coli to major hormones and polyamines present in human gut
Source: ISME J. 2018 Jul 11;12(11):2736–47. doi: 10.1038/s41396-018-0227-5 (PMC6194112; doi:10.1038/s41396-018-0227-5)
Supplement: Supplementary file 11 — Table S2 [file 41396_2018_227_MOESM11_ESM.pdf]

Table S2: Strains and plasmids used in this study

	Relevant genotype	Induction	Source
**Strains**			
MG1655	*E. coli* K12	_	(Blattner *et al* 1997)
RP437	*E.coli* K12 derivative; wild type for chemotaxis	_	(Parkinson and Houts 1982)
VS104	*E. coli* RP437 Δ(*cheY cheZ*)	_	(Sourjik and Berg 2002)
VS181	*E. coli* RP437 Δ(*cheY cheZ*) Δ(*tar, tsr, trg, tap, aer*)	_	(Sourjik and Berg 2004)
VS188	*E. coli* RP437 Δ(*tar, tsr, trg, tap, aer*)		(Ames *et al* 2002)
Δ*trg*	*E. coli* RP437 Δ (*cheR-cheZ*) trg::Tn10	_	(Kalinin *et al* 2010)
Δ*tap*	*E. coli* RP437 Δ(*cheY cheZ*) tap	_	(Sourjik and Berg 2004)
ΔpotA	*E. coli* BW25113 Δ*potA*::Km^r	_	(Baba *et al* 2006)
ΔpotD	*E. coli* BW25113 Δ*potD*::Km^r	_	(Baba *et al* 2006)
**Plasmids**			
pVS88	CheY-EYFP / CheZ-EYFP expression plasmid, pTrc99a derivative, Amp^R	50 µM IPTG	(Sourjik and Berg 2004)
pVS1092	Tar expression plasmid, pKG110 derivative, Cam^R	2.0 µM salicylate	(Yang *et al* 2015)
pPA114	Tsr expression plasmid, pKG110 derivative, Cam^R	0.7 µM salicylate	(Ames *et al* 2002)
pKG116	Expression vector; p15A ori, derivative of pACYC184, Cam^R	1 µM salicylate	J.S. Parkinson
pJL02	PotD expression plasmid, pKG116 derivative, Cam^R	1 µM salicylate	This study

Ames P, Studdert CA, Reiser RH, Parkinson JS (2002). Collaborative signaling by mixed chemoreceptor teams in *Escherichia coli*. Proc Natl Acad Sci U S A 99: 7060-7065.

Baba T, Ara T, Hasegawa M, Takai Y, Okumura Y, Baba M *et al* (2006). Construction of *Escherichia coli* K-12 in-frame, single-gene knockout mutants: the Keio collection. Mol Syst Biol 2: 2006 0008.

Blattner FR, Plunkett G, 3rd, Bloch CA, Perna NT, Burland V, Riley M *et al* (1997). The complete genome sequence of *Escherichia coli* K-12. Science 277: 1453-1462.

Kalinin Y, Neumann S, Sourjik V, Wu M (2010). Responses of *Escherichia coli* bacteria to two opposing chemoattractant gradients depend on the chemoreceptor ratio. J Bacteriol 192: 1796-1800.

Parkinson JS, Houts SE (1982). Isolation and behavior of *Escherichia coli* deletion mutants lacking chemotaxis functions. J Bacteriol 151: 106-113.

Sourjik V, Berg HC (2002). Receptor sensitivity in bacterial chemotaxis. Proc Natl Acad Sci U S A 99: 123-127.

Sourjik V, Berg HC (2004). Functional interactions between receptors in bacterial chemotaxis. Nature 428: 437-441.

Yang Y, A MP, Hofler C, Poschet G, Wirtz M, Hell R *et al* (2015). Relation between chemotaxis and consumption of amino acids in bacteria. Mol Microbiol 96: 1272-1282.
